# Embedding Research on Emotion Duration in a Network Model

**DOI:** 10.1007/s42761-023-00203-3

**Published:** 2023-08-12

**Authors:** Jens Lange

**Affiliations:** University of Hamburg, Von-Melle-Park 5, 20146 Hamburg, Germany

**Keywords:** Emotion duration, Network model, Emotion theory, Emotion coherence, Formal model

## Abstract

Contrary to early theorizing, emotions often last for longer periods of time. Variability in people’s emotion duration contributes to psychopathologies. Therefore, emotion theories need to account for this variability. So far, reviews only list predictors of emotion duration without integrating them in a theoretical framework. Mechanisms explaining why these predictors relate to emotion duration remain unknown. I propose to embed research on emotion duration in a network model of emotions and illustrate the central ideas with simulations using a formal network model. In the network model, the components of an emotion have direct causal effects on each other. According to the model, emotions last longer (a) when the components are more strongly connected or (b) when the components have higher thresholds (i.e., they are more easily activated). High connectivity prolongs emotions because components are constantly reactivated. Higher thresholds prolong emotions because components are more easily reactivated even when connectivity is lower. Indirect evidence from research on emotion coherence and research on the relationship of predictors of emotion duration with components outside of emotional episodes supports the usefulness of the network model. I further argue and show in simulations that a common cause model, in which a latent emotion causes changes in emotion components, cannot account for research on emotion duration. Finally, I describe future directions for research on emotion duration and emotion dynamics from a network perspective.

**Supplementary Information:**

The online version contains supplementary material available at 10.1007/s42761-023-00203-3.

Emotions are commonly defined as relatively *short-term* experiences (e.g., Ekman & Cordaro, 2011; Keltner & Gross, 1999). However, evidence indicates that the duration of emotional episodes is highly variable (Frijda et al., 1991; Verduyn et al., 2012). Differences between people in how long their (negative) emotions last are indicative of psychopathological states (e.g., Houben et al., 2015; Lapate & Heller, 2020). Hence, emotion theories need to account for varying patterns of emotion duration. Reviews primarily list predictors of emotion duration without integrating the findings into a broader theoretical framework (Verduyn, 2021; Verduyn et al., 2015). Mechanisms underlying the relation between these predictors and emotion duration remain largely unknown. My goal is to provide an integrative theoretical framework of emotion duration by embedding it in a network model of emotions.

## Emotion Duration

Initially, theorizing argued that emotions are short-term experiences (e.g., Ekman, 1992). Emotions synchronize people’s feelings, cognition, physiology, motivation, and expression in response to personally relevant situations (e.g., Keltner & Gross, 1999; Levenson, 1994). The entire process was argued to last only a few seconds (e.g., Ekman, 1999; Ekman & Cordaro, 2011). However, studies soon showed that actual emotional episodes often last for minutes or even hours (Sonnemans & Frijda, 1994; Verduyn et al., 2012).

Notably, there is substantial variability between persons, emotions, or episodes of the same emotion in how long the episode lasts. Accordingly, research started investigating predictors of emotion duration, identifying multiple characteristics of the person, the emotion, or the emotion-eliciting event that predict the duration of an emotional episode (Table 1; for comprehensive reviews, see Verduyn, 2021; Verduyn et al., 2015). For instance, people higher in neuroticism or rumination have longer negative emotional episodes, people higher in depression have longer negative and shorter positive emotional episodes, people higher in extraversion have longer positive emotional episodes, and people higher in trait reappraisal or who currently reappraise the situation have shorter positive and negative emotional episodes. Furthermore, emotional episodes are longer when they are intense and the emotion-eliciting event is highly relevant.

**Table 1:** Predictors of Emotion Duration And Their Potential Relation to Network Connectivity And Component Thresholds

Predictor	Relation to Emotion Duration	Relation to Network Connectivity (suggested by emotion coherence research)	Relation to Component Thresholds (suggested by component-focused research)
Neuroticism	↑ negative emotions	↓ for negative emotions	↑ for negative emotions
Extraversion	↑ positive emotions	? for positive emotions	↑ for positive emotions
Depression	↑ negative emotions; ↓ positive emotions	↓ for negative and positive emotions	↑ for negative emotions; ↓ for positive emotions
Rumination	↑ negative emotions	? for negative emotions	↑ for negative emotions
(Trait) Reappraisal	↓ for negative and positive emotions	↓ or 0 for negative and positive emotions	↓ for negatives and positive emotions
Emotion Intensity	↑ for negative and positive emotions	↑ for negative and positive emotions	cannot be determined
Event Relevance	↑ for negative and positive emotions	↑ for negative emotions; ? for positive emotions	↑ for negative and positive emotions

↑ implies that relation is positive. ↓ implies that relation is negative. ? implies that relation is unclear

Compiling lists of central predictors is of utmost importance. However, the mechanisms explaining why these predictors relate to emotion duration remain unknown. Embedding these findings in a theoretical framework would go beyond specific predictors toward a more general understanding of the dynamics of emotions (Muthukrishna & Henrich, 2019). It may also provide intervention strategies for dealing with problematic emotion duration in psychopathologies. I argue that embedding research on emotion duration in a network model of emotions promises to advance the field.

## Emotion Duration in a Network Model of Emotions

**Fig. 1:**
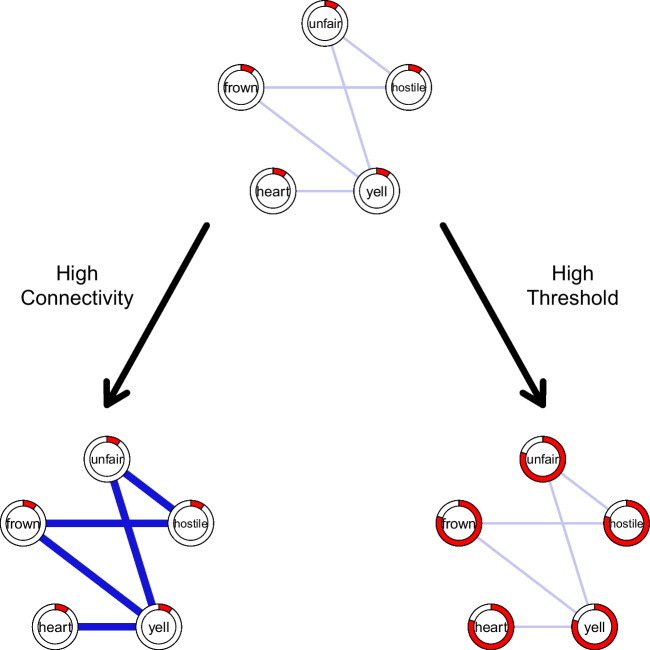
An Emotion Network And It’s Characteristics Affecting Emotion Dynamics. *Note.* The top network represents a simplified network of an anger episode. The nodes represent components of anger and the relationships represent positive causal relationships between the components. The bottom networks illustrate the two network characteristics affecting the dynamics of an emotion, namely higher connectivity (i.e., average strength of the causal relationships; illustrated with stronger relationships) and higher thresholds (i.e., the state in which the component tends to be; illustrated with the filling level of the border of a component). unfair – cognitive appraisal of unfairness, hostile – hostile feelings, yell – motivation to yell, heart – accelerated heart rate, frown – frowning

The central idea of a network model of emotions is that the components of an emotion have direct causal effects on each other (Lange et al., 2020; Lange & Zickfeld, 2021; see also Suri & Gross, 2022). When visualized, these causal relationships look like a network. For instance, a person may perceive an insult as unfair, leading to hostile feelings and to the motivation to yell, for which the body mobilizes energy by accelerating heart rate and starting to frown, while frowning feeds back into feelings via facial feedback. The entire episode represents *anger* (Fig. 1). The more components of anger are activated, the more intense the emotional experience is. Importantly, there is no monolithic network structure for a particular emotion. Instead, there is variability between persons and situations (Lange et al., 2020).

Research on other kinds of psychological networks indicates that variability in two independent characteristics of a network critically affect its dynamics (Fig. 1): (a) *connectivity*—how strongly the components are connected—and (b) each component’s *threshold*—the state in which a component tends to be (e.g., Cramer et al., 2016; Dalege et al., 2016). When conceptualizing emotional episodes as networks of causal relationships, the same characteristics should affect the dynamics of emotions.

If the components of an emotion’s network are more strongly connected, activation of one or more component(s) also more quickly activates other components of the network. Once the network is active, its activity is sustained by reactivation via feedback loops between components. Thus, higher connectivity will prolong the duration of an emotional episode.

Moreover, a component’s threshold indicates in which state the component tends to be. When the threshold is high, the component tends to be active and can, hence, be more easily activated all else being equal. For instance, for components of anger, does a person often consider situations unfair or tends to yell a lot? If thresholds of a network are high, its components will be more easily activated and also more easily reactivated even when the network’s connectivity is lower. Thus, high component thresholds will also prolong the duration of an emotional episode.

To specify and explore these ideas, I varied the two characteristics in a formal network model (inspired by Cramer et al., 2016; Lunansky et al., 2020; technical details are available in the Supplementary Materials and code is on OSF). To base the formal model on realistic emotion networks, I took network structures from another study. Specifically, I used common methods for estimating networks from data when researchers aim to investigate theoretical causal network models (for a discussion of the limitations of these methods, see Lange & Zickfeld, 2021). I estimated two networks with data from a study in which participants rated sets of components of *awe* and *fear* after watching movies of threatening natural events (e.g., tornados; Lange and Zickfeld, in press). I then simulated an external event that activates all components of the respective network (e.g., watching a movie) and that then faded over arbitrary units of time as if the emotional person is occupied with the event for some time. Once the external activation vanished, the emotion will return to its baseline at some point. The first return to baseline is a straightforward definition of the end of an emotional episode (Verduyn, 2021). I tracked the intensity of the emotion as the mean activity of all components. For instance, when seven of 10 components are active, the emotion’s intensity is 0.7. Once the intensity after the event, was no longer significantly larger than the mean intensity prior to the event, the emotion ended. In separate simulations, I varied the respective network’s connectivity or thresholds.

**Fig. 2:**
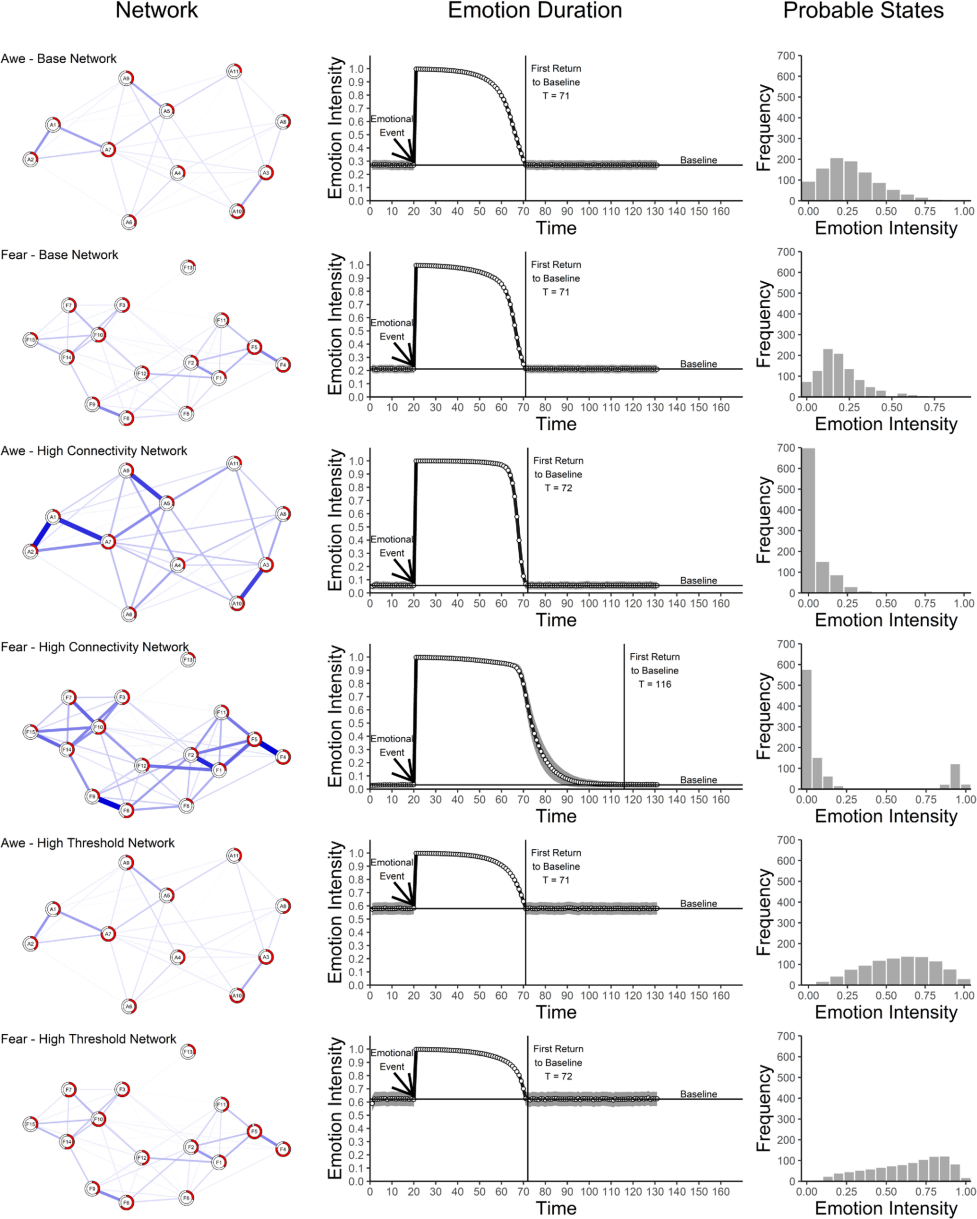
Illustration of The Central Ideas With Simulated Data. *Note.* Base networks were estimated with data from Lange and Zickfeld (in press). Each node in the network represents an item measuring one of the components of the respective emotion, while the filling level of the border illustrates the estimated threshold. Blue/red relationships represent positive/negative relationships between the components, while thicker relationships represent stronger relationships. To generate networks with higher connectivity, relationships were multiplied with a factor. To generate networks with higher thresholds, a constant was added to the thresholds. For each network, the second column shows simulated data for the duration of an emotional episode with such a network. The white dots, connected by a black line, show the mean intensity of the emotion. The grey area around the line represents ± 25 *SE* of the mean intensity across multiple runs of the simulation. The *Baseline* represents the mean emotion intensity prior to the emotion-eliciting event. The *First Return to Baseline* represents the first emotion intensity that is no longer significantly larger than the Baseline (i.e., the end of the emotional episode). For each network, the third column shows simulated data for probable states one would encounter over multiple persons or situations for such a network. Details on the data set, formal model, and the simulations are available in the Supplementary Materials. A – *awe*, F – *fear*, T – Time

The simulations corroborated the theorizing (Fig. 2), although the results are stronger for *fear* than for *awe*. Both higher connectivity (for both emotions) and higher thresholds (only for *fear*) led to higher emotion duration. For higher connectivity, the network also kept its high-intensity state for longer. Beyond these primary findings, higher thresholds also resulted in higher baseline intensity because the components tend to be active. In contrast, higher connectivity even reduced the baseline intensity because the initially inactive components keep each other in check.

An alternative model inspired by affect program theories (Levenson, 1994; for a similar argument, see Coan, 2010) fails to account for research on emotion duration (full description of the model and simulation results reported in the Supplementary Materials). In this model, there is a common cause that affects all emotion components, while the components have no causal relationships among each other. Independent of the strength of relationships between the common cause and the components, the emotion always ends once the external activation vanished because the components cannot reactivate each other. This finding complements other evidence that people’s self-rated experiences of emotions are best captured by a network model as compared to such alternative models (Lange and Zickfeld, in press).

### Predictors of Emotion Duration, Network Connectivity, and Thresholds

Fully embedding research on emotion duration in a network model of emotions requires evidence that predictors of emotion duration relate to network connectivity and component thresholds. Such research is nonexistent. However, there is indirect evidence from two separate lines of research. First, the notion of network connectivity is reminiscent of the notion of emotion coherence. Emotion coherence indicates how strongly the component changes of an emotion (e.g., feelings and expressions of anger) are correlated in an emotional episode. Notably, there is substantial variability between persons and situations in how strongly emotions cohere (e.g., Mauss & Robinson, 2009). If predictors of emotion duration are also predictors of emotion coherence, then research on emotion coherence provides indirect evidence that these predictors relate to differences in network connectivity.

Second, the component changes of an emotion can occur in non-emotional situations. For instance, people differ in how unfairly they tend to perceive situations or in how frequently they yell also in situations in which they do not experience anger. Akin to thresholds, people would therefore more easily experience such a component all else being equal. If evidence indicates that predictors of emotion duration are also predictors of general tendencies for component changes outside of emotional episodes, then this research provides indirect evidence that these predictors relate to differences in thresholds.

Indeed, the two lines of research provide indirect evidence that predictors of emotion duration relate to differences in network connectivity and component thresholds (Table 1). Neuroticism predicted less coherence of facial expressions and physiology in sadness (Wu et al., 2021), yet neuroticism predicted higher thresholds for various components associated with many negative emotions (Kuppens & Tong, 2010) such as lower autonomy, more worrying, and being pessimistic (Soto & John, 2017) as well as being motivated to avoid (Smits & Boeck, 2006). For extraversion, no research linked it to emotion coherence of positive emotions, yet it predicted higher thresholds for components associated with many positive emotions such as higher control, being assertive, higher power, and being active (Soto & John, 2017) as well as being motivated to approach (Smits & Boeck, 2006). Depression predicted less coherence of various components for both positive and negative emotions (Bendezú et al., 2022; Brown et al., 2020; Kahn et al., 2021; Mauss et al., 2005; Sommerfeldt et al., 2019) and predicted higher thresholds for components associated with many negative emotions such as inactivity or loss of interest (American Psychiatric Association, 2000) as well as a cognitive style predisposing to experience negative emotions (Hankin et al., 2005). For rumination, no research linked it to emotion coherence of negative emotions, yet it predicted higher thresholds for components associated with many negative emotions such as a maladaptive cognitive style and lower motivation to act (Nolen-Hoeksma et al., 2008) as well as appraisals of intrusive thoughts about the likelihood of challenging relevant situations (Watkins, 2004). (Trait) reappraisal had a mixed relationship with emotion coherence for various components for both positive and negative emotions (Brown et al., 2020; Butler et al., 2014), yet it, by definition, predicts a lower threshold of appraisals involved in an emotional episode because the activation of appraisals is attenuated or opposing appraisals are activated.

Similar findings exist for research on characteristics of the emotional situation. Emotion intensity predicted higher emotion coherence of various components for both positive and negative emotions (Bonanno & Keltner, 2004; Brown et al., 2020; Mauss et al., 2005; Rosenberg & Ekman, 1994; Schaefer et al., 2014). The relationship of emotion intensity and thresholds cannot be determined because emotional intensity cannot occur outside of an emotional episode. Finally, event relevance predicted more coherence of various components for negative emotions (Calvo & Miguel-Tobal, 1998; Lohani et al., 2018), while there is no research on emotion coherence for positive emotions, and event relevance also predicted a higher threshold for components associated with positive and negative emotions such as perceiving potentially emotion-eliciting cues more quickly, and a higher motivation to prioritize, think about, and respond to such cues (Klinger & Cox, 2004).

Thus, different predictors may change emotion duration via different mechanisms. Neuroticism, depression, and (trait) reappraisal may primarily predict differences in thresholds as implied by research on their relationship with emotion components outside emotional episodes. In contrast, they may predict network connectivity, if anything, negatively, as implied by research on their relationship with emotion coherence. Even though evidence is less strong, the same conclusions may apply to extraversion and rumination. Emotion intensity and event relevance may further positively predict network connectivity as these predictors also positively relate to emotion coherence.

## Future Research Directions

Beyond providing a theoretical framework for embedding research on emotion duration, the approach also makes new predictions. First, as shown in Fig. 2, depending on the mechanism, the shape of the intensity profile of an emotional episode should differ. If a predictor relates to higher emotion duration via higher connectivity (emotion intensity, event relevance), the intensity profile should show an abrupt decrease, while the intensity should remain rather high throughout. This pattern results because strongly connected components prefer to be in the same state, either active or inactive. Hence, they will tend to be all activated as long as possible and once the external activation becomes low enough, they will all tend to deactivate quickly. If a predictor relates to higher emotion duration primarily via higher thresholds (neuroticism, extraversion, depression, rumination), their intensity profile should show a more steady decrease over time. For lower thresholds (reappraisal), the decrease should be quicker because the components tend to be inactive and therefore strive to reach that state quicker. Some evidence supports these predictions (Heylen et al., 2015; Verduyn et al., 2012).

Second, depending on the mechanism, a predictor may also relate to other patterns of emotion dynamics (for background, see Cramer et al., 2016; Dalege et al., 2016; Gilmore, 1981). As shown in Fig. 2, predictors relating to higher connectivity should predict higher variance in emotion intensity before the end of an emotional episode because the highly interconnected components reactivate each other from time to time. Furthermore, they should predict lower variance in emotion intensity outside of emotional episodes because components keep each other in check. For predictors relating to higher thresholds, variance outside emotional episodes is even higher.

Moreover, depending on the mechanism, one should encounter different emotional states. That is, different states are probable. Imagine asking a person multiple times how intensely they currently experience *fear* and tracking how often they experience each level of intensity. Or imagine asking multiple persons in only one situation and tracking how many persons experience each level of intensity. Both these approaches result in frequencies of different emotional intensities. Different people or situations lead to different frequencies (Haslbeck et al., 2023) and these differences may result from differences in the underlying networks. Hence, different predictors of emotion duration may also result in different frequencies of probable states. As is also shown in Fig. 2, for predictors relating to higher connectivity one should encounter especially low and high intensity states, whereas for predictors relating primarily to differing thresholds one should encounter many high intensity states with a skewed tail of low intensity states. Relatedly, intervening on network characteristics (e.g., Blanken et al., 2019; Haslbeck et al., 2021) may allow shaping people’s emotion dynamics, helping them to deal with problematic emotion duration in psychopathologies. For instance, regulating a strongly connected component should have a larger effect on emotion duration than regulating a weakly connected component. Future research should test these patterns directly and intervene on them.

One limitation, however, is that the evidence relating predictors of emotion duration to network connectivity and thresholds is only indirect. Therefore, before investigating additional patterns, research should determine emotion networks separately for different persons or situations using tools from network science (e.g., Borsboom et al., 2021; Frewen et al., 2012; Lange & Zickfeld, 2021, Lange and Zickfeld, in press) and relate the predictors to network connectivity and thresholds directly. Complementarily, future research could extend and explore the formal network model used for the simulations (see Supplementary Materials for details and avenues for future research).

Moreover, contrary to predictions, higher thresholds did not lead to higher emotion duration for the *awe* network. Potentially, the *awe* network featured too many weak relationships between components. Then, even if the components tend to be active, the network cannot sustain its active state with reactivation via feedback loops. I tested this reasoning in another simulation reported in the Supplementary Materials. Indeed, increasing thresholds in the high connectivity *awe* network led to higher emotion duration.

Finally, the causal direction of various relationships is unknown. For instance, maybe neuroticism causes changes in thresholds. However, maybe neuroticism *is* a state defined by differences in thresholds (Cramer et al., 2012) or emotional processes cause changes in neuroticism (Lunansky et al., 2020). Relatedly, in line with evidence that various relationships between components of emotions are bidirectional (Scherer & Moors, 2019) and that components of an emotion are only partly connected (Mauss & Robinson, 2009), I used partly connected, bidirectional networks estimated from data for the simulations. However, in actual networks, at least some relationships may be in only one direction, which could be investigated with directed acyclic graphs (for an accessible introduction, see Rohrer, 2018). Moreover, the networks may have specific structures (e.g., a small world; Lange et al., 2020). Thus, research should clarify the causal role of predictors of emotion duration as embedded in even more realistic causal network models.

## Conclusion

People often experience emotions over extended periods of time, while various characteristics of the person, the emotion, or the emotion-eliciting event predict variability in emotion duration. I argued that all the predictors may relate to emotion duration via only two mechanisms: network connectivity and component thresholds. By embedding research on emotion duration in a network model, I thereby integrated scattered findings and paved the way towards new research on emotion dynamics.

### Supplementary Information

Below is the link to the electronic supplementary material.

Supplementary file1 (DOCX 1327 KB)

## Additional Information

### Data availability

Data and code are available on the Open Science Framework (https://osf.io/mbhjn/).

### Code availability

Data and code are available on the Open Science Framework (https://osf.io/mbhjn/).
