# Supplementary material for: Embedding Research on Emotion Duration in a Network Model
Source: Affect Sci. 2023 Aug 12;4(3):541–9. doi: 10.1007/s42761-023-00203-3 (PMC10513999; doi:10.1007/s42761-023-00203-3)

**Embedding Research on Emotion Duration in a Network Model**

Jens Lange

University of Hamburg

Jens Lange
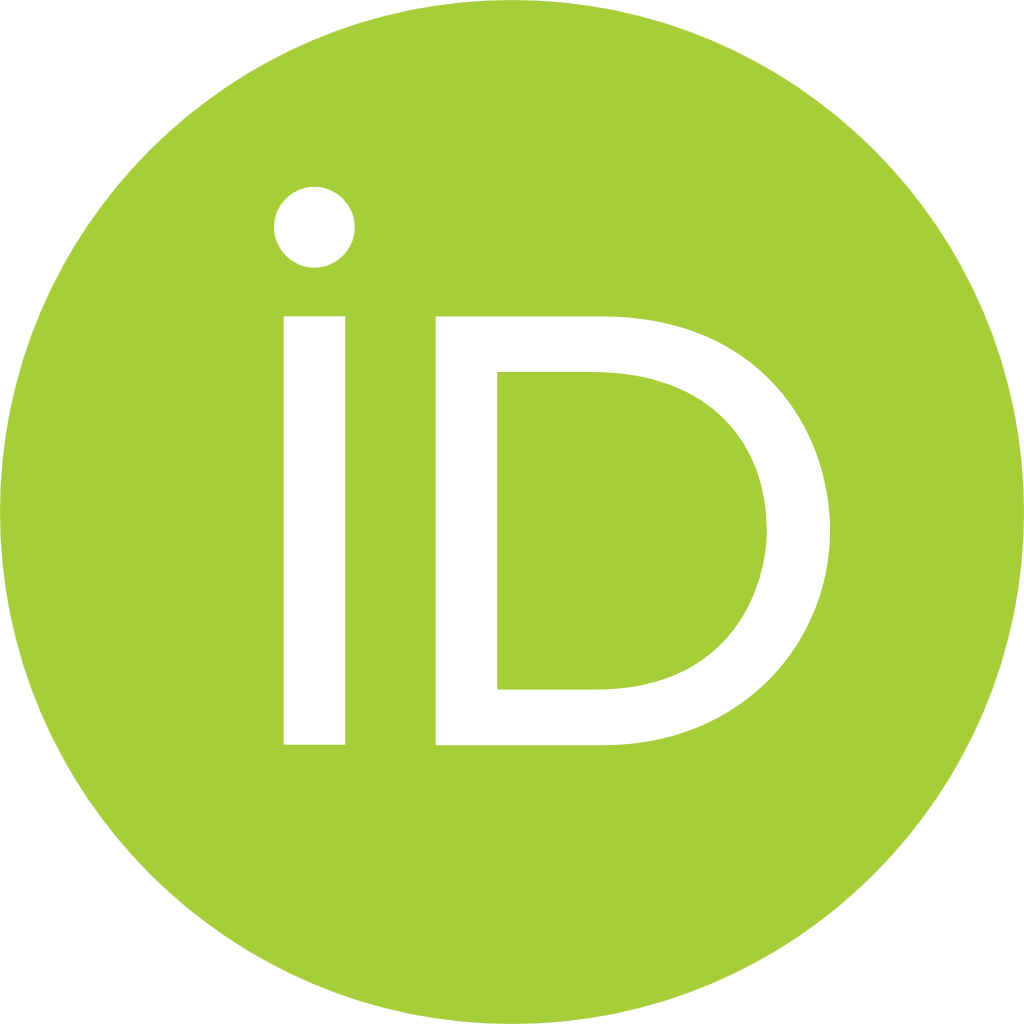
https://orcid.org/0000-0002-5375-3247

Correspondence should be addressed to Jens Lange, University of Hamburg, Von-Melle-Park 5, 20146 Hamburg, Germany, E-mail: [lange.jens@outlook.com](mailto:lange.jens@outlook.com).

journal: *Affective Science*

**Supplementary Materials**

**The Formal Network Model**

To illustrate how variability in network connectivity and component thresholds affect emotion duration and can lead to new predictions for emotion dynamics, I programmed a formal network model based on the Ising model (Cramer et al., 2016; Lunansky et al., 2020; see also Ising, 1925). I programmed the model as a function in *R* (R Core Team, 2019; version 4.2.3), using the packages *igraph* (Csárdi & Nepusz, 2006; version 1.4.1), *Matrix* (Bates et al., 2022; version 1.5-3), *qgraph* (Epskamp et al., 2012; version 1.9.4), *bootnet* (Epskamp et al., 2018; version 1.5), and *lavaan* (Rosseel, 2012; version 0.6-15). Hence, users can load the function into *R* and, by changing the function arguments, they can adapt and run the model for their research purposes. Moreover, users can extend the function if certain functionality is missing. The model is available on OSF in version 4, which resulted from changes from previous versions (simulateEmotionModel4.R). The *R* file also includes extensive documentation in the form of comments.

The central parts of the model are the components of an emotion and their bi-directional relationships. The components can be either on (1) or off (0). During the simulations, the states are coded as 1 and -1 (see Haslbeck et al., 2020 for a discussion of codings of the Ising model) and transformed back to 1 and 0 in the output for ease of presentation. Each relationship has a certain edge weight, coding how strongly positive/negative it is.

Users can set a number of arguments. Most importantly, they can specify an emotion model. Users can plug in an existing network that they estimated from other data or that they created in a different way. Moreover, they can also create networks of their preferred size and edge weights with a small-world structure (Watts & Strogatz, 1998), a preferential-attachment structure (Barabási & Albert, 1999), or a random structure (Erdös & Renyi, 1959). Moreover, for each network, users can increase the network’s connectivity, by specifying a multiplier for the average edge weight, and set the threshold of each component.

With the specified network, users can run two different kinds of simulations. First, users can simulate how the network would react to varying external activation. This kind of simulation could be used for exploring the duration of an emotional episode for such a network. Users specify a vector of numbers representing multiple time points with different external activation. During the simulation, for each time point, the external activation is added to the user-specified thresholds. The idea is that higher/lower external activation increases/decreases the probability of a component to be activated. For the network at each time point, the model then uses Glauber dynamics (Glauber, 1963) to simulate a likely state of each component. Depending on users’ preference, the model then stores the average activity of the network at each time point as the mean of the states of all components or the activity of each component separately. The simulation can be repeated multiple times by choosing a specific number of the function argument *k*. The output of this kind of simulation includes the weights matrix of the specified or simulated network model and a data frame with one row for each specified value of external activation and *k* columns for each estimated mean emotion intensity or *k* times the number of components columns for the estimated states of each component.

Second, users can simulate 1,000 probable states the specified network could take. This kind of simulation could be used for exploring what kind of states one would encounter for such a network. Based on the specified network, the model generates probable states of an Ising model. Depending on users’ preference, the model then also re-estimates the network model with the 1,000 simulated states, allowing to test whether the specified network parameters can be recovered from the simulated data. The simulation can again be repeated *k* times. The output of this kind of simulation includes the weights matrix of the specified or simulated network model and a data frame with one row for each possible state the network could take (i.e., from zero components activated to all components activated) and *k* columns with the frequencies with which each state was encountered. Finally, if requested, the output also includes a list with all the re-estimated networks.

**Details on The Reported Simulations With The Network Models**

I used the formal network model to illustrate the central ideas. I conducted simulations for emotion duration and probable states of two emotion networks by varying their connectivity and thresholds. I conducted the simulations in *R* using the packages *psych* (Revelle, 2018; version 2.3.3), *ggplot2* (Wickham, 2016; version 3.4.1), *officer* (Gohel, 2022; version 0.6.2), and *future.apply* (Bengtsson, 2021; version 1.10.0) next to the packages required for the formal model.

To conduct the simulation, I used two existing networks. Doing so ensures that the networks used in the simulation have a structure that is likely for emotion networks. Specifically, I estimated two networks, one for *awe* and one for *fear*, using data originally published in Lange and Zickfeld (in press). Participants in this study watched short movies of natural disasters (e.g., tornados) known to elicit both emotions simultaneously. They then answered how much items measuring components of both emotions applied to them on a scale from 1 (*not at all*) to 7 (*very much*). Table S1 reports the items and descriptive statistics.

**Table S1**

*Descriptive Statistics of the Items*

| Label | Item | *M* (*SD*) |
| --- | --- | --- |
| Awe | | |
| A1 | I felt my jaw drop. | 2.31 (1.62) |
| A2 | I gasped. | 2.19 (1.62) |
| A3 | I felt that I was in the presence of something grand. | 4.63 (1.86) |
| A4 | I sensed things momentarily slow down. | 2.92 (1.76) |
| A5 | I had goosebumps. | 2.55 (1.73) |
| A6 | I felt challenged to mentally process what I was experiencing. | 2.87 (1.76) |
| A7 | I felt my eyes widen. | 3.50 (1.85) |
| A8 | I had the sense of being connected to everything. | 3.33 (1.75) |
| A9 | I had chills. | 2.57 (1.73) |
| A10 | I felt awed. | 4.75 (1.82) |
| A11 | I felt that my sense of self was diminished. | 2.48 (1.74) |
| Fear | | |
| F1 | I felt afraid. | 2.56 (1.69) |
| F2 | I felt nervous. | 2.63 (1.71) |
| F3 | I was out of control. | 1.57 (1.20) |
| F4 | I could imagine a disastrous outcome of such a situation. | 5.04 (1.75) |
| F5 | I perceived such a situation to be dangerous. | 5.05 (1.84) |
| F6 | My heart beat faster. | 2.80 (1.73) |
| F7 | I had moist hands. | 1.46 (0.92) |
| F8 | My muscles tensed up. | 2.30 (1.55) |
| F9 | My breathing got faster. | 2.39 (1.57) |
| F10 | I was weak in the knees. | 1.58 (1.05) |
| F11 | I wanted to quickly get out of such a situation. | 2.74 (1.90) |
| F12 | I was speechless. | 2.65 (1.74) |
| F13 | I attended to everything around me. | 2.52 (1.70) |
| F14 | I had a trembling voice. | 1.33 (0.80) |
| F15 | I let out a yell. | 1.24 (0.73) |

*Note*. Participants provided answers on a scale from 1 (*not at all*) to 7 (*very much*).

I estimated two Ising models, one for each emotion. To this end, I binarized the variables such that all values with the scale midpoint of 4 and lower indicated that a component was off (0) and all values above 4 indicated that the component was on (1). For five items (F3, F7, F10, F14, and F15), the item difficulty was so high, that I had to binarize them at 2. I then estimated the networks with the *IsingFit* default settings in the *bootnet* package, excluding missing values listwise, and applying the *OR*-rule.

For the simulation of emotion duration, I specified a vector of external activations that should mimic a classic emotional event. Initially, there was zero activation for 20 time points to establish a baseline. Then, an emotional event abruptly occurred which raised the thresholds by 2.5. Subsequently, the external activation faded in steps of 0.05 per time point until it reached zero again. The idea was that the emotional event preoccupied the person for some time but slowly faded. Hence, at Time 71, the emotional event had completely faded (i.e., external activation is zero again). Finally, there were 60 time points with zero external activation to test when the emotion activity would return to baseline and thereby end the emotional episode. For each time point, I stored the mean emotion intensity. I marked the point at which the emotional episode ended as the first time point at which the 95% CI of mean intensity over all iterations of the simulation included the mean of the first 20 time points that established a baseline.

In the simulations, I separately varied connectivity and thresholds. Specifically, the simulation had a 2 emotion (*awe* vs. *fear*) X 3 kind of network (base vs. high connectivity vs. high threshold) design. For connectivity, I multiplied the relationships with 2.25. I settled on this value because then the edge weights were not too extreme to be unrealistic and the results illustrated the point I tried to make. For thresholds, I added 0.3 to all estimated thresholds. I settled on this value for similar reasons as for connectivity. I ran all simulations *k* = 10,000 times and averaged over these 10,000 simulations.

For the simulation of probable states, I just plugged in the networks and simulated data from them. I used the same settings for higher connectivity and higher thresholds as before. I ran all simulations only *k* = 45 times because there is way less variation in the distribution of probable states as each simulation already simulates 1,000 states. For each simulation, I stored the frequencies with which all states occurred. I then averaged over all 45 simulations.

**Simulation With Higher Thresholds in The High Connectivity *Awe* Network**

Contrary to predictions, higher thresholds did not lead to higher emotion duration in the *awe* network in the simulation reported in the main text. When looking at the distribution of edge weights of the *awe* and *fear* networks, it appears that the *awe* network has fewer strong relationships. I suspected that higher thresholds may lead to higher emotion duration only a network features a high number of strong relationships. Only in such a network, the activity of components that tend to be active can be sustained by feedback loops. To test this logic, I ran a simulation with the same settings as in the simulation reported in the main text, in which I increased the thresholds in the high connectivity *awe* network. As the increase by 0.3, which I used in the simulations in the main text when increasing the thresholds, supported the prediction but also led to a weird result, I reran the simulation with a smaller increase of 0.2. The results are presented in Figure S2.

The weird result remained. Unexpectedly, the mean intensity increased slightly over the first few time points even though the external activation remained 0 throughout these steps. I assume that this weird result stems from the fact that in the simulation all components are initially inactive. The high connectivity forces all components to be in the same state, which would typically lead to an inactive network, as is shown for the high connectivity *awe* network with lower thresholds. The higher thresholds, however, move the baseline activity up. Potentially, it requires a few time steps to reach a stable state.

To avoid that the weird result affects conclusions about the central hypothesis, I just excluded these initial time points from the calculation of the baseline before the event. Including these time steps would have substantially lowered the baseline, preventing that the emotion ever ends. This finding would have supported the hypothesis that higher thresholds in the high connectivity network lead to higher emotion duration, yet this confirmatory evidence would be biased by the weird result. Still, even when excluding the first seven time points from the calculation of the baseline, the hypothesis is supported. Thus, in line with my reasoning, higher thresholds lead to higher emotion duration even in the *awe* network, if there are more strong relationships.

**An Alternative Model – The Common Cause Model**

There are various conceptualizations of emotions next to conceptualizing them as networks. Prominently, in line with affect program theories, emotions may actually be common causes of their components (Levenson, 1994; for a similar perspective on affect program theories, see Coan, 2010; Russell, 2003). That is, a personally relevant situation activates a central mechanism, which then elicits all components of an emotion, while the components have no direct causal effects among each other (see also Figure S1). Research indicates that this model cannot account for various findings related to emotions (Lange et al., 2020; Lange & Zickfeld, in press). However, can it account for research on emotion duration?

I argue that it cannot. In fact, in a strict sense, the common cause model cannot provide a mechanism that leads to variance in emotion duration. The components are all activated only by the latent emotion. And the latent emotion is activated by external activation. Once the external activation fully vanished, the latent emotion is no longer activated, and, by extension, also the components are no longer activated. Put differently, in the common cause model, the emotion always ends approximately when the emotional event is over, because the components cannot reactivate each other.

If anything, it is imaginable that the latent emotion somehow remains activated once the external activation fully vanished. Then, it could, by extension, also further activate the components. Such a model would require that there is a separate mechanism that keeps the latent emotion activated. Such a mechanism, however, has not been proposed as far as I know. Thus, in the most popular depiction of the common cause model, it cannot account for research on emotion duration.

To illustrate this idea, I extended the formal model in *R* by the option to also simulate varying activation and probable states for the common cause model, either by creating a new common cause model or by plugging in an existing one. Note that the common cause model is reminiscent of the reflective latent variable model, in which a latent variable causes changes in its indicators (for a discussion, see Lange et al., 2020). Therefore, in the formal model, users can also specify to investigate the reflective latent variable model. When doing so, the model gets another part, namely the latent emotion. The latent emotion serves as a common cause of the components such that the model includes directed relationships from the latent emotion to the components. User can set the weights of these relationships as for the network models. Moreover, they can set a multiplier for the average weight as strength (akin to connectivity for networks). As before, components can be on (1) or off (0), while internally the states are coded 1 and -1. Finally, the components also have thresholds the user can specify as for the network models.

From this model, users can also simulate the exposure to varying external activation. To this end, users have to specify a vector with numbers of external activation as before. For each component, the external activation of a time point is multiplied with the weight of the relationship between the latent emotion and the respective component. The probability of being active for a component is then $p=1 / (1+e^{\left( -t-a \right)})$, in which $p$ is the probability of being active, $t$ is the threshold of the respective component, and $a$ is the activation received from the latent emotion. Using a random experiment, the model then determines for each component whether it will be active. The output is then the same as for the network models, except that the reflective latent variable model as specified in *lavaan* is part of the output instead of the network weights matrix.

Users can also simulate 1,000 probable states. To this end, the model simulates data from the reflective latent variable model in *lavaan*. It treats the model as multivariate normal, meaning that all components take a state on a normal distribution with *M* = 0 and *SD* = 1. To binarize the values, all values above the user-specified threshold are coded as on and below the threshold as off. As for the network models, users can also request that the simulated data is used to re-estimate the model. The output is then the same as for the network models, again except that the model syntax is part of the output instead of the network weights matrix.

I used the formal model to simulate varying activation and probable states for the reflective latent variable model. I decided against fitting a reflective latent variable model to the *awe* and *fear* data and using these models to run the simulations. Evidence indicates that the reflective latent variable model does not fit to this data set (Lange & Zickfeld, in press). Using these data-based models as starting points for the simulation would have, hence, been an unfair test for the common cause model. A fair test requires that the theoretical notion of the common cause model is implemented in the model from which one simulates data. Thus, I created realistic common cause models and used these for the simulation.

The model had 12 components that were caused by an emotion with an average relationship of *M* = 0.5 and *SD* = 0.05. I set the thresholds to -0.5 similar to the network models. As for the network models, for the high strength model, I increased the strength to 2.25, and for the high threshold model, I increased the threshold by 0.3. The vector of external activations was the same as for the network models. For the simulation of varying activation, I again simulated *k* = 10,000 runs and for the simulation of probable states I again simulated *k* = 45 runs.

The results are presented in Figure S1. As predicted, all models had exactly the same emotion duration supporting that the model cannot account for research on emotion duration. Emotional episodes ended at the end of external activation at Time 71. They also all had a steady decline of emotion intensity. The only differences were that the high strength model led to a stronger increase after the emotional event and the high threshold model led to higher baseline emotional intensity. For the probable states, the data are approximately normally distributed for the base model and the high threshold model. For the high strength model, the distribution becomes extremely right skewed.

**Potential Extensions of The Formal Model**

Next to the future research directions outlined in the manuscript, future research may also explore and extend the formal model more systematically. I want to propose four possibilities. First, it would be interesting to investigate the emotion duration of specific components of a network, which the formal model already allows. Some components last longer than others (Verduyn et al., 2015). The network model predicts that more strongly connected components will last longer because they are more easily re-activated during an emotional episode, as long as their thresholds are not particularly low as compared to the other components. Relatedly, intervening on more strongly connected components is then also a plausible intervention strategy to increase or decrease emotion duration in problematic cases. For instance, reappraisal is potentially more effective as a regulation strategy than suppression also because appraisals are more strongly connected in a network than facial expressions. Relatedly, one could also extend the formal model such that the external activation activates only one of the components of the network (e.g., the cognitions) instead of all of them.

Second, it would be interesting to investigate the effect of different network structures, which users can already specify in the model. The simulation results were stronger for *fear* than for *awe*. Potentially, the *awe* network features less strongly connected cliques of interconnected components, thereby reducing the probability that different emotion components reactivate each other. Relatedly, users could estimate networks of different emotions and compare their dynamics in the formal model, which may align with differences in emotion duration for different emotions identified in previous research (Verduyn et al., 2015).

Third, future research should explore the role of the external activation in predicting the duration and shape of emotion intensity of an emotional episode. I used a vector of values of external activation that abruptly increased the external activation after a short period that established a baseline. Subsequently, the external activation slowly faded. Therefore, all simulated emotional episodes started abruptly and then the intensity decreased steadily over time. However, emotional episodes also vary in how abruptly they start and whether they have multiple peaks (Verduyn et al., 2009). Reproducing such patterns or making new predictions about how connectivity and thresholds affect them requires testing the influence of different vectors of values of external activation.

Finally, it would be interesting to extend the formal model with formal models representing other emotion theories. For instance, from a constructionist perspective, emotions can be conceptualized as causal effects of their components, reversing the logic of the common cause model (e.g., Coan, 2010). This model is akin to the formative latent variable model. In such a model, there are relationships between different components that may reactivate each other, yet the components are external to the emotion itself (Lange et al., 2020). It is therefore difficult to predict the dynamics of an emotion in the formative model. To explore these dynamics, future research may implement the option to simulate from a formative model.

References

Barabási, A.-L., & Albert, R. (1999). Emergence of scaling in random networks. *Science*, *286*(5439), 509–512. https://doi.org/10.1126/science.286.5439.509

Bates, D., Maechler, M., & Jagan, M. (2022). *Matrix: Sparse and dense matrix classes and methods*. https://CRAN.R-project.org/package=Matrix

Bengtsson, H. (2021). A unifying framework for parallel and distributed processing in R using futures. *The R Journal*, *13*(2), 208. https://doi.org/10.32614/RJ-2021-048

Coan, J. A. (2010). Emergent ghosts of the emotion machine. *Emotion Review*, *2*(3), 274–285. https://doi.org/10.1177/1754073910361978

Cramer, A. O. J., van Borkulo, C. D., Giltay, E. J., van der Maas, H. L. J., Kendler, K. S., Scheffer, M., & Borsboom, D. (2016). Major depression as a complex dynamic system. *PLOS ONE*, *11*(12), e0167490. https://doi.org/10.1371/journal.pone.0167490

Csárdi, G., & Nepusz, T. (2006). The igraph software package for complex network research. *Inter Journal Complex Systems*, *1695*, 1–9.

Epskamp, S., Borsboom, D., & Fried, E. I. (2018). Estimating psychological networks and their accuracy: A tutorial paper. *Behavior Research Methods*, *50*, 195–212. https://doi.org/10.3758/s13428-017-0862-1

Epskamp, S., Cramer, A. O. J., Waldorp, L. J., Schmittmann, V. D., & Borsboom, D. (2012). qgraph: Network visualizations of relationships in psychometric data. *Journal of Statistical Software*, *48*(4). https://doi.org/10.18637/jss.v048.i04

Erdös, P., & Renyi, A. (1959). On random graphs. *Publicationes Mathematicae*, *6*, 290–297.

Glauber, R. J. (1963). Time‐dependent statistics of the Ising Model. *Journal of Mathematical Physics*, *4*(2), 294–307. https://doi.org/10.1063/1.1703954

Gohel, D. (2022). *officer: Manipulation of Microsoft Word and PowerPoint documents*. https://CRAN.R-project.org/package=officer

Haslbeck, J. M. B., Epskamp, S., Marsman, M., & Waldorp, L. J. (2020). Interpreting the Ising Model: The input matters. *Multivariate Behavioral Research*, 1–11. https://doi.org/10.1080/00273171.2020.1730150

Ising, E. (1925). Beitrag zur Theorie des Ferromagnetismus. *Zeitschrift für Physik*, *31*(1), 253–258. https://doi.org/10.1007/BF02980577

Lange, J., Dalege, J., Borsboom, D., Van Kleef, G. A., & Fischer, A. H. (2020). Toward an integrative psychometric model of emotions. *Perspectives on Psychological Science*, *15*(2), 444–468. https://doi.org/10.1177/1745691619895057

Lange, J., & Zickfeld, J. H. (in press). Comparing implications of distinct emotion, network, and dimensional approaches for co-occurring emotions. *Emotion*. https://doi.org/10.1037/emo0001214

Levenson, R. W. (1994). Human emotions: A functional view. In P. Ekman & R. J. Davidson (Eds.), *The nature of emotions: Fundamental questions* (pp. 123–126). Oxford University Press.

Lunansky, G., Borkulo, C., & Borsboom, D. (2020). Personality, resilience, and psychopathology: A model for the interaction between slow and fast network processes in the context of mental health. *European Journal of Personality*, *34*(6), 969–987. https://doi.org/10.1002/per.2263

R Core Team. (2019). *R: A language and environment for statistical computing.* R Foundation for Statistical Computing. https://www.R-project.org/

Revelle, W. (2018). *psych: Procedures for personality and psychological research*. Northwestern University. https://CRAN.R-project.org/package=psych

Rosseel, Y. (2012). lavaan: An R package for structural equation modeling. *Journal of Statistical Sofware*, *48*(2), 1–36.

Russell, J. A. (2003). Core affect and the psychological construction of emotion. *Psychological Review*, *110*(1), Article 1. https://doi.org/10.1037/0033-295X.110.1.145

Verduyn, P., Delaveau, P., Rotge, J.-Y., Fossati, P., & Van Mechelen, I. (2015). Determinants of emotion duration and underlying psychological and neural mechanisms. *Emotion Review*, *7*(4), 330–335. https://doi.org/10.1177/1754073915590618

Verduyn, P., Van Mechelen, I., Tuerlinckx, F., Meers, K., & Van Coillie, H. (2009). Intensity profiles of emotional experience over time. *Cognition & Emotion*, *23*(7), 1427–1443. https://doi.org/10.1080/02699930902949031

Watts, D. J., & Strogatz, S. H. (1998). Collective dynamics of ‘small-world’ networks. *Nature*, *393*(6684), 440–442. https://doi.org/10.1038/30918

Wickham, H. (2016). *ggplot2: Elegant graphics for data analysis*. Springer.

**Fig. S1**

*Illustration of an Alternative Model With a Central Cause With Simulated Data*

*Note.* Base models were created as to be realistic. The latent variable (circle) represents an unobservable (i.e., latent) emotion. Each indicator (square) in the model represents a component of the emotion, while the filling level of the border illustrates the threshold. Blue/red relationships represent positive/negative relationships between the emotion and the components, while thicker relationships represent stronger relationships. To generate a model with higher strength, relationships were multiplied with a factor. To generate a model with higher thresholds, a constant was added to the thresholds. For each model, the second column shows simulated data for the duration of an emotional episode with such a model. The white dots, connected by a black line, show the mean intensity of the emotion. The grey area around the line represents ±25 *SE* of the mean intensity across multiple runs of the simulation. The *Baseline* represents the mean emotion intensity prior to the emotion-eliciting event. The *First Return to Baseline* represents the first emotion intensity that is no longer significantly larger than the Baseline prior to the emotion-eliciting event (i.e., the end of the emotional episode). For each model, the third column shows simulated data for probable states one would encounter over multiple persons or multiple situations for the same person with such a model. C – Component, T – Time.





**Fig. S2**

*Illustration of The Effect of Higher Thresholds in The High Connectivity* Awe *Network With Simulated Data*

*Note.* The network was estimated with data from Lange and Zickfeld (in press). Each node in the network represents an item measuring *awe*, while the filling level of the border illustrates the estimated threshold. Blue/red relationships represent positive/negative relationships between the components, while thicker relationships represent stronger relationships. To generate the networks with higher connectivity, relationships were multiplied with a factor. To generate networks with higher thresholds, a constant was added to the thresholds. For each network, the second column shows simulated data for the duration of an emotional episode with such a network. The white dots, connected by a black line, show the mean intensity of the emotion. The grey area around the line represents ±25 *SE* of the mean intensity across multiple runs of the simulation. The *Baseline* represents the mean emotion intensity prior to the emotion-eliciting event. The *First Return to Baseline* represents the first emotion intensity that is no longer significantly larger than the Baseline (i.e., the end of the emotional episode). For each network, the third column shows simulated data for probable states one would encounter over multiple persons or situations for such a network. A – *awe*, T – Time.


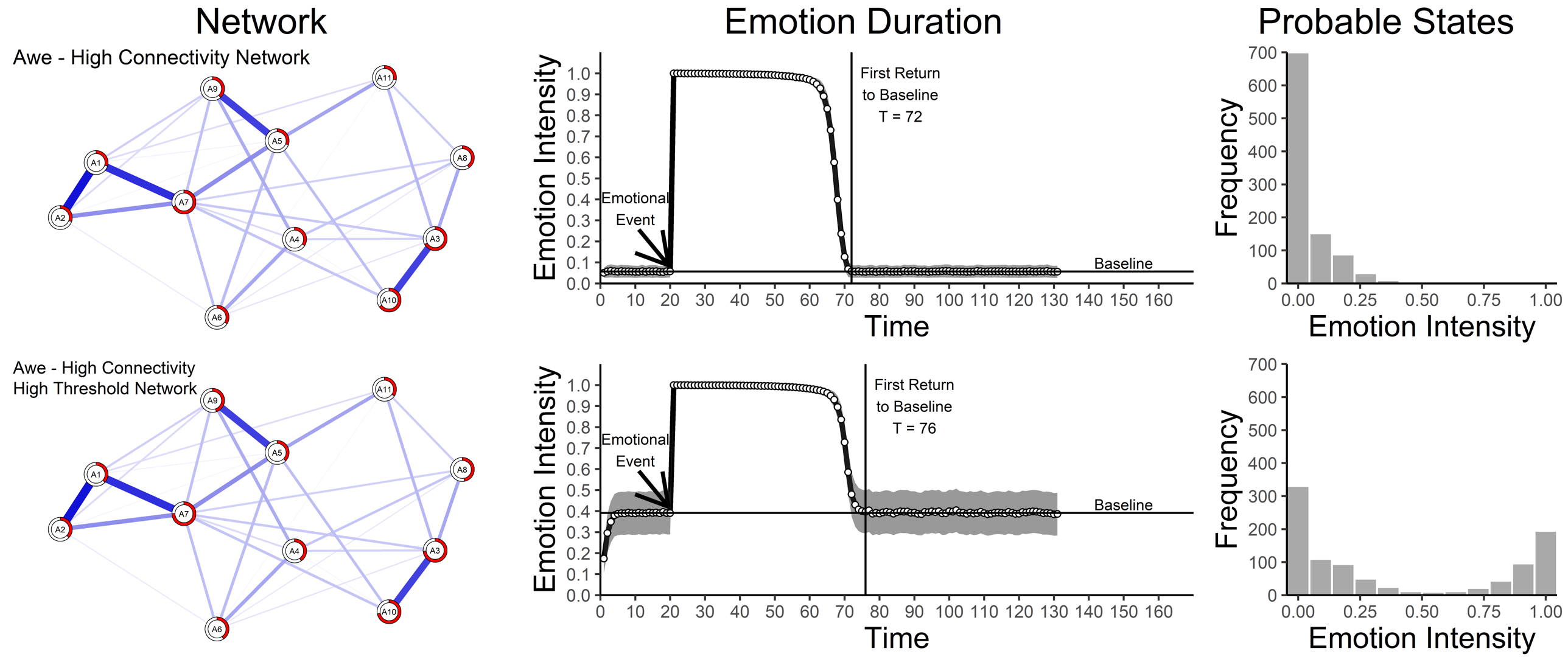

Supplement: Supplementary file 1 — Supplementary file1 (DOCX 1327 KB) [file 42761_2023_203_MOESM1_ESM.docx]
